# Letter from the Editor in Chief

**DOI:** 10.19102/icrm.2020.111101

**Published:** 2020-11-15

**Authors:** Moussa Mansour


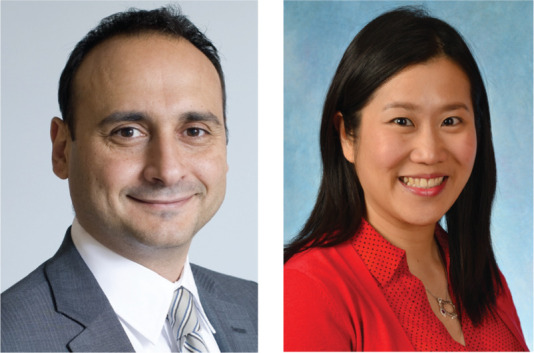


Dear Reader,

The 15^th^ Annual Symposium on Ventricular Arrhythmias, held last month in a virtual format, covered a wide range of topics, including the mapping of intramural ventricular tachycardia (VT) and ectopy, which was discussed extensively and is expected to gain momentum in the next few years.

Dr. Fermin García described the value of combining epicardial mapping of the coronary venous system, intramural mapping of septal perforator venous branches, and endocardial mapping for the treatment of left ventricular premature beats (PVCs), which assists in localizing the layer of origin of the PVCs and successfully guiding radiofrequency energy delivery to the optimal site.

Similarly, Dr. Frank Bogun described outcomes of the ablation of focal intramural ventricular ectopy by targeting the site of origin in 83 patients with intramural PVCs. Here, a stepwise approach of coronary-venous-system venography and perforator-vein mapping to confirm the site of origin, followed by unipolar RF delivery at the site of origin if it was reachable or from an adjacent site if it could not be reached by the ablation catheter, was adopted. The site of origin was identified in 19 patients and ablation performed in these areas eliminated PVCs in all cases. A success rate of 67% was achieved using ablation targeting the endocardial breakout sites in the remaining 64 patients.

Meanwhile, Dr. William Stevenson discussed ablation outcomes in 20 patients with periaortic VT, in whom bipolar voltage was reduced relative to in the normal population but not in a manner different from in patients with nonperiaortic VT. Unipolar voltage, however, was lowest in the periaortic VT group, suggesting intramural scar. An aggressive ablation strategy including needle ablation with or without simultaneous unipolar ablation resulted in a success rate of 80%.

Finally, Dr. Usha Tedrow discussed the substrate characterization of intramural VT, suggesting that the live substrate from intramural VT can be identified by a frequency band of a unipolar recording from the needle catheter. In this context, the intramural excitable scar signal decreased to the same level as the transmural scar signal after needle ablation.

Ventricular ectopy and tachycardia can be a challenging clinical scenario; however, the innovative mapping and ablation techniques presented at the 2020 VT Symposium are likely to facilitate procedural success in this regard.

Best regards and we hope that you enjoy reading this issue of *The Journal of Innovations in Cardiac Rhythm Management*.

Sincerely,


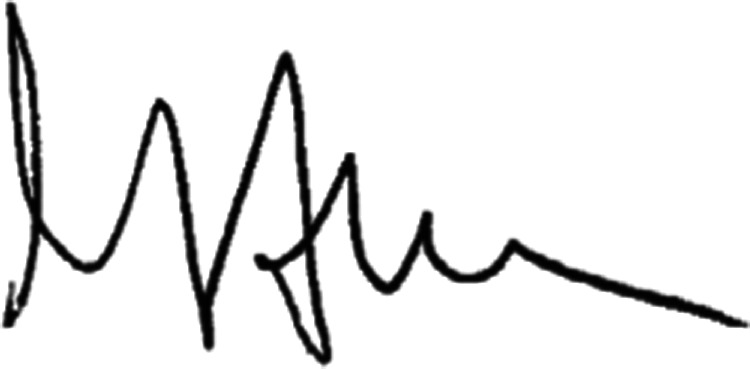


Moussa Mansour, MD, FHRS, FACC

Editor in Chief

The Journal of Innovations in Cardiac Rhythm Management

MMansour@InnovationsInCRM.com

Director, Atrial Fibrillation Program

Jeremy Ruskin and Dan Starks Endowed Chair in Cardiology

Massachusetts General Hospital

Boston, MA 02114

and

Weeranun Bode, MD

Cardiac Electrophysiology Fellow

Massachusetts General Hospital

Boston, MA 02114

